# Cellulose acetate membranes loaded with combinations of tetraphenylporphyrin, graphene oxide and Pluronic F-127 as responsive materials with antibacterial photodynamic activity†

**DOI:** 10.1039/d3ra04193j

**Published:** 2023-09-07

**Authors:** Rania E. Morsi, Denis Gentili, Franco Corticelli, Vittorio Morandi, Alberto Figoli, Francesca Russo, Francesco Galiano, Giovanna Angela Gentilomi, Francesca Bonvicini, Ilse Manet, Barbara Ventura

**Affiliations:** a Egyptian Petroleum Research Institute (EPRI) PO Box 11727 Nasr City Cairo Egypt; b Institute for Organic Synthesis and Photoreactivity (ISOF), National Research Council (CNR) Via P. Gobetti 101 40129 Bologna Italy barbara.ventura@isof.cnr.it ilse.manet@isof.cnr.it; c Institute of Nanostructured Materials (ISMN), National Research Council (CNR) Via P. Gobetti 101 40129 Bologna Italy; d Institute for Microelectronics and Microsystems (IMM), National Research Council (CNR) Via P. Gobetti 101 40129 Bologna Italy; e Institute on Membrane Technology (ITM), National Research Council (CNR) Via P. Bucci 17/C 87036 Rende (CS) Italy; f Department of Pharmacy and Biotechnology, University of Bologna Via Massarenti 9 40138 Bologna Italy; g Microbiology Unit, IRCCS Azienda Ospedaliero-Universitaria di Bologna Via Massarenti 9 40138 Bologna Italy

## Abstract

The development of polymeric fabrics with photoinduced antibacterial activity is important for different emerging applications, ranging from materials for medical and clinical practices to disinfection of objects for public use. In this work we prepared a series of cellulose acetate membranes, by means of phase inversion technique, introducing different additives in the starting polymeric solution. The loading of 5,10,15,20-tetraphenylporphyrin (TPP), a known photosensitizer, was considered to impart antibacterial photodynamic properties to the produced membranes. Besides, the addition of a surfactant (Pluronic F-127) allowed to modify the morphology of the membranes whereas the use of graphene oxide (GO) enabled further photo-activated antibacterial activity. The three additives were tested in various concentrations and in different combinations in order to carefully explore the effects of their mixing on the final photophysical and photodynamic properties. A complete structural/morphologycal characterization of the produced membranes has been performed, together with a detailed photophysical study of the TPP-containing samples, including absorption and emission features, excited state lifetime, singlet oxygen production, and confocal analysis. Their antibacterial activity has been assessed *in vitro* against *S. aureus* and *E. coli*, and the results demonstrated excellent bacterial inactivation for the membranes containing a combination of the three additives, revealing also a non-innocent role of the membrane porous structure in the final antibacterial capacity.

## Abstract 2

Cellulose acetate (CA) membranes loaded with a photosensitizer, 5,10,15,20-tetraphenylporphyrin (TPP), a surfactant and graphene oxide (GO) display effective photoinactivation of colonies of both *S. aureus* and *E. coli*.

## Graphical abstract

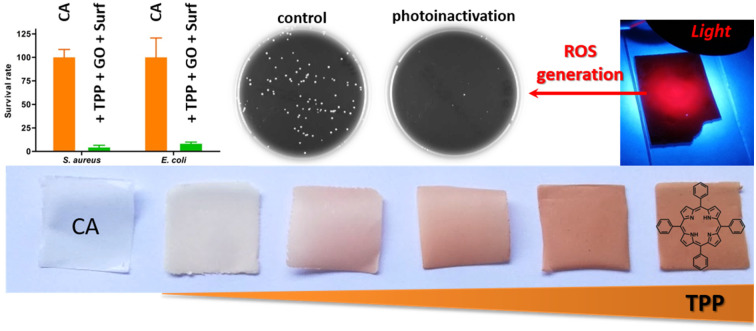

## 1. Introduction

The rise of bacterial infections caused by antibiotic-resistant pathogens is one of the most serious problems for public health worldwide.^1^ To overcome this problem there is an urgent need for new technologies and innovative approaches prompting researchers to search for antimicrobial solutions free from antibiotics. In this context, the development of safe and efficient bactericidal materials represents a highly valuable tool. Antimicrobial surfaces, in particular, are among the most appealing materials, since they can find application in wound dressings, medical device disinfection, and treatment of objects in public environments.^2,3^

Different approaches exist to confer bactericidal properties to a surface, among which photodynamic inactivation of bacteria is gaining interest. The latter is an emerging strategy to treat drug-resistant microbes. It relies on the properties of a photosensitizer that, upon absorption of light, through population of its triplet excited state reacts with oxygen, generating singlet oxygen (type II photosensitization) or other reactive oxygen species (ROS, type I photosensitization) that lead to bacteria inactivation. The advantages of the treatment reside on its spatial and temporal control, the use of a clean energy source such as light, its repeatability without cumulative toxicity and its efficacy towards clinically relevant multidrug resistant bacteria.^4^ In this perspective, the incorporation of a photosensitizer into polymeric fabrics, either membranes^5,6^ or fiber-based materials, is a promising strategy to create solid scaffolds with photoinduced antibacterial activity. The short lifetime of the produced reactive species (few μs) requires closeness between the photosensitizer and the bacterial cell, so that the porosity of the material is an important parameter to be considered in the design of efficient scaffolds.

Polymers are materials of choice in membrane technology thanks to the flexibility and scalability of the fabrication process. Phase inversion, which consists in removing the solvent from a polymer solution in a way that leaves a porous solid membrane, is the most commonly used process for membranes fabrication. Interestingly, membranes with different pore dimensions and networks can be obtained by introducing modifications in the fabrication conditions.^7^ Among the different polymers used for membrane fabrication, cellulose acetate combines source availability, biocompatibility, low cost and simple processing.^8^

The use of carbon-based materials, graphene oxide (GO) in particular, is emerging in membrane research, since these additives have shown to impart robustness and improved mechanical properties to the polymeric scaffolds.^9,10^ On the other hand, GO is also widely explored in nanomedicine, thanks to its unique chemical and electrochemical properties and its ability to combine with other nanoscale materials.^11,12^ Interestingly, GO is explored as an antimicrobial agent able to destroy the bacterial cell membrane, but its efficacy in this respect is somehow controversial.^13–16^ Its antibacterial activity is also associated with a photothermal mechanism, where a localized hyperthermia produced upon light irradiation in the Vis-NIR region leads to bacterial damage.^16–20^

Thanks to these promising characteristics, the combination of photosensitizers with GO is an emerging approach to develop materials with improved photoinduced antibacterial properties.^21–23^

In this work, a series of cellulose acetate membranes was fabricated by means of phase inversion technique, introducing modifications in the preparation conditions. The addition of a surfactant to the polymeric solution was explored in order to modify the porous structure of the membrane, while the loading of GO was introduced to impart both structural and functional properties to the produced material. The desired antibacterial activity has been further addressed with the loading of a photosensitizer (5,10,15,20-tetraphenylporphyrin, TPP) in the polymeric matrix. Different loadings of GO and TPP and different combinations of the chosen additives were probed in order to identify possible synergic/disfavouring effects. The produced membranes were fully characterized in terms of cross-section morphology, surface roughness, pore size and porosity, photophysical properties and singlet oxygen production capacity. Eventually, the antibacterial activity of the membranes was assessed *in vitro* against two model strains, *Staphylococcus aureus* and *Escherichia coli*, and the results have been discussed in relation to the structural/photophysical properties of the porous photoactive scaffolds.

## 2. Materials and methods

### 2.1. Materials

The following chemicals were used as received: cellulose acetate (average *M*_n_: 30 000), Pluronic F-127, 5,10,15,20-tetraphenylporphyrin (TPP), 5,10,15,20-tetrakis(4-sulfonatophenyl)-porphyrin (TPPS_4_) and anthracene-9,10-dipropionic acid disodium salt (ADPA) from Sigma-Aldrich, anhydrous DMF (max 0.005% water) from VWR Chemicals, sodium chloride (for analysis) from Carlo Erba.

### 2.2. Basic membrane preparation

Cellulose acetate (CA) membranes were prepared by phase inversion technique, using 20% CA solution in DMF and using an automatic film applicator (Automatic Film Applicator Compact AB3655, from TQC Sheen) with controlled thickness of the casted solution and speed of film spreading. The solution was prepared by stirring 20 wt% CA in DMF overnight using a magnetic stirrer. The produced homogeneous solution was carefully casted on a plate fixed on the film applicator surface and then spread using an applicator moving with a pre-set thickness at a fixed speed (500 μm and 5 mm s^−1^, respectively). After spreading, the plate with the produced casted solution was smoothly immersed into a deionized water bath. Within few time, the casted film got solidified and turned from transparent to opaque, then the produced membrane got separated from the glass plate. After complete separation, the membrane was then immersed in another fresh deionized water bath overnight. The membranes produced with this procedure were stored in deionized water to keep their shape, physical and mechanical properties. Before characterization, the membranes were removed from water and dried under vacuum at room temperature.

### 2.3. Preparation of modified membranes

#### 2.3.1. Modification with surfactant

In this modification we explored the addition of a surfactant to the polymer solution before fabrication. In the experiment, 5% (weight% surfactant/polymer) of Pluronic F-127 was mixed with the polymer solution using a magnetic stirrer. The solution mixture was then fabricated as described in the basic procedure. The produced membrane was abbreviated as CA_SURF.

#### 2.3.2. Modification with graphene oxide (GO)

Different weight percentages of GO with respect to the polymer were dispersed in DMF, added to the polymer solution and homogenized prior to membrane fabrication. The solution mixture was then fabricated as described in the basic procedure. Three membranes were produced with 0.005%, 0.01% and 0.05% GO percentages, abbreviated below as CA_0.005% GO, CA_0.01% GO and CA_0.05% GO, respectively.

#### 2.3.3. Modification with the photosensitizer (TPP)

TPP was dissolved in DMF and added to the polymer solution before fabrication. A homogeneous solution of the polymer and TPP was obtained by continuous stirring overnight of the mixture and then degassing the solution by passing N_2_ gas for 20 min to remove any air bubbles raised during the mixing process. Different loadings of TPP (weight% TPP/polymer) have been explored: 0.005%, 0.1%, 0.5% and 1%. The 0.1% loading was chosen to be combined with: (i) the addition of the surfactant, (ii) the addition of GO 0.01% and (iii) the addition of both the surfactant and GO 0.01% (with the surfactant always added as first additive and TPP as last additive).

Casted films obtained with the polymer solution containing TPP 0.05% and 0.1% have been also prepared for comparison purposes in the photophysical analysis of the membrane samples.

### 2.4. Membrane characterization

#### 2.4.1. Membranes morphology

The cross-section morphologies of the prepared membranes were investigated using Scanning Electron Microscopy (SEM, LEO 1530 FEG model). The cross section was prepared using liquid nitrogen to stiffen the sample before breaking; after that, the sample was coated with a thin gold layer (about 10 nm thickness).

#### 2.4.2. Membranes surface analysis

Atomic Force Microscopy (AFM) images were collected in air on a Multimode8 microscope operated in Peak Force mode and equipped with a type J scanner (Bruker Nano Inc. GmbH, Berlin, Germany). Background interpolation and surface roughness parameter calculations were performed with Gwyddion 2.48 (http://gwyddion.net/).

#### 2.4.3. Pore size and pore size distribution

Pore size and pore size distribution were investigated by a Capillary Flow Porometer (POROLUXTM 1000, Porometer, IB-FT GmbH, 12277 Berlin, Germany). A fluorinated test liquid (Porewick®) was used as wetting liquid (surface tension: 16 dynes per cm).

#### 2.4.4. Porosity

The measurement of the porosity was performed by initially weighting three different parts (big, medium and small) from each membrane after soaking them into water. The weight of the three parts was measured again once they were dried by keeping them in the oven for 24 hours at 40 °C.

The membrane porosity was calculated by means of the following eqn (1):1
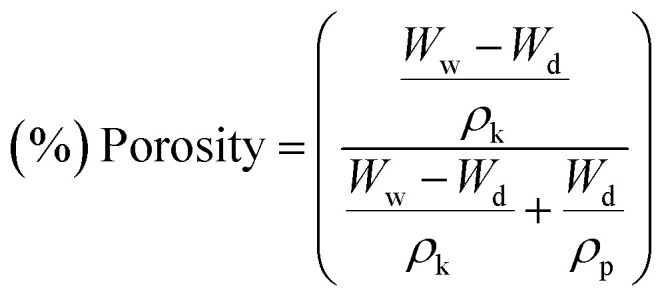
where *W*_w_ is the weight of the wet membrane, *W*_d_ is the weight of the dried membrane, *ρ*_k_ is the density of water (0.997 g cm^−3^) and *ρ*_p_ is the polymer density (1.305 g cm^−3^).

#### 2.4.5. Photophysical characterization and confocal analysis

Photophysical measurements were performed on membrane squares of 25 × 25 mm placed between two quartz slides. Reflectance spectra were acquired with a PerkinElmer Lambda 950 UV/vis/NIR spectrophotometer equipped with a 100 mm integrating sphere and converted in absorption spectra using the Kubelka–Munk function.^24^ Emission spectra were collected in front-face mode with an Edinburgh FLS920 fluorimeter equipped with a Peltier-cooled Hamamatsu R928 PMT (280–850 nm) and, when indicated, corrected for the wavelength dependent phototube response. Fluorescence lifetimes were measured by using an IBH time-correlated single-photon counting (TCSPC) apparatus by using a pulsed NanoLED excitation source at 465 nm. Analysis of the luminescence decay profiles against time was accomplished with the Decay Analysis Software DAS6 provided by the manufacturer. Averaged values from different batches of the same sample and from collection at both 650 nm and 720 nm for each sample have been considered. Estimated errors are 10% on lifetimes and 2 nm on emission and absorption peaks.

Singlet oxygen measurements were performed by placing the membrane on the inner wall of a spectrophotometric quartz cuvette and filling the cuvette with 3 mL of a solution of ADPA in water (*ca.* 6.0 × 10^−5^ M). The membrane was then irradiated at 512 nm, for incremental time intervals, by using an irradiation setup composed by a 150 W Xenon lamp (LOT) and an Omni-λ150 monochromator (Zolix) completed by a 375 nm cut-off filter, under continuous stirring of the solution. The irradiated spot size was the same for all experiments. The light intensity was 0.15 mW cm^−2^. ADPA is a commonly used singlet oxygen probe to measure the singlet oxygen production of photosensitizers in aqueous solutions. It reacts with singlet oxygen yielding an endoperoxide with concomitant decrease of its absorbance in the 320–400 nm region, that can be monitored spectrophotometrically.^25–27^ Noticeably, in our setup the measurement takes place in heterogeneous conditions, as the TPP, producing the singlet oxygen upon irradiation, is embedded in the membrane, while ADPA is in solution. A control experiment has been conducted in homogeneous conditions by preparing, in the dark, a solution containing ADPA 6.0 × 10^−5^ M and TPPS_4_ 4.6 × 10^−6^ M and irradiating it at 512 nm with the same experimental setup.

Fluorescence confocal imaging was performed on an inverted Nikon Ti-E microscope (Nikon Co., Shinjuku, Japan). The confocal fluorescence microscope Nikon A1 is equipped with an Argon ion CW laser and 640 nm CW diode laser as well as 405 nm and 637 nm pulsed/CW diode lasers (PicoQuant GmbH, Berlin, Germany). Images were collected using a Nikon Plan Apo VC 20× objective with NA 0.9. Filters were set to register fluorescence in the 560–630 nm range and the 660–740 nm range.

Spectral imaging was performed with the Nikon spectral module of 32 PMT setting a range of 10 nm per detector and the membranes were excited either at 488 nm or 405 nm.

Fluorescence lifetime imaging was performed by exciting with the pulsed 405 or 637 nm diode laser and collecting photons with integrated PicoHarp 300 electronics (PicoQuant GmbH, Berlin, Germany) for TCSPC measurements. A single-photon avalanche diode detector equipped with a 695–735 nm bandpass filter was used as detector. Histograms of collected photons consist in 3200 channels each with 16 ps width. The repetition rate of the pulsed excitation was 20 MHz. The instrument response function of the system is approximately 220 ps. The fluorescence decay fit was performed on the histogram calculated for a region of interest. The fluorescence decay profile was analyzed with a least-squares method, using biexponential decay function provided by Picoquant SymPhoTime software.

The fitting function used is:

2 *I*(*t*) = *b* + *Σ*_j_*a*_j_ exp(−*t*/*τ*_j_)

The fractional intensity and the average fluorescence lifetime are calculated according to the following equations:

3 *f*_i_ = *a*_i_*τ*_i_/*Σ*_j_*a*_j_*τ*_j_

4 *τ*_av_ = *Σ*_j_*f*_j_*τ*_j_

#### 2.4.6. Antibacterial activity

The *in vitro* photoinactivation efficacy of the fabricated membranes was determined by using *Staphylococcus aureus* (ATCC 25923) and *Escherichia coli* (ATCC 25922) as Gram-positive and Gram-negative model systems, respectively. The reference strains were purchased from the American Type Culture Collection (ATCC) and routinely cultured in 5% blood agar plate at 37 °C. For experiments, bacterial suspensions were prepared in PBS (phosphate buffer saline), adjusted at 0.5 McFarland, corresponding to 10^8^ CFU (colony forming units) per mL.

Samples of the herein devised membranes (Ø = 0.6 cm) were individually placed into wells of a 48-well plate and incubated with 100 μL of the prepared suspensions for 1 hour at 37 °C in the dark to allow bacterial deposition and strictly adhesion to the membrane surfaces. After this period, samples were exposed for 30 minutes to a continuous light delivered from a 100 W mercury lamp (C-SHG1, Nikon corp., Japan), equipped with a 540/25 nm filter. The light intensity was 43 mW cm^−2^. Two controls were included in each experiment: a light control consisting of the unmodified CA membrane submitted to the same irradiation procedure, and a dark control being made of the modified membrane but kept in the dark.

Once illuminated, bacterial suspensions and membranes were collected, vortexed to re-suspend and detach bacteria from the membranes, and serially diluted in PBS. Each sample was spread on plate count agar (Liofilchem s.r.l, Italy) for the determination of the number of viable bacteria. After 24 hours of incubation at 37 °C, plates were imaged using the VersaDoc Image System, and colonies were digitally counted in the most convenient dilution using the colony counting software (Quantitation Software Version 4.6.9, Quantity One, Bio-Rad Laboratories, Inc., Hercules, CA, USA).

#### 2.4.7. Data analysis

The potency of the antibacterial photodynamic activity was expressed in terms of survival rate that is the percentage value of the CFUs of the illuminated sample *vs.* the corresponding dark control. Differences between samples were determined by unpaired Student's *t* test, or one-way ANOVA followed by Dunnett's Multiple Comparison Test using the GraphPad Prism Software, version 6.0 (GraphPad Software). Statistical significance was set at *p* values < 0.05.

## 3. Results and discussion

### 3.1. Design and fabrication of the membranes

The development of the CA membranes was achieved by using phase inversion in controlled conditions, in terms of thickness of the casted solution and speed of the film spreading. In this technique, a casted polymer solution is subjected to deionized water as a non-solvent. Under these conditions, the polymer starts to be thermodynamically unstable and separate into a polymer-rich phase and a polymer-poor phase, induced by the presence of the non-solvent. At this point, the system can be described as a ternary system consisting of the polymer, the solvent and the non-solvent. This stage is followed by the replacement of the solvent with the non-solvent, which results in the solidification of the polymer and formation of a porous membrane.^28^

As described in the experimental section, the basic procedure has been varied introducing different additives in the polymeric solution prior to membrane fabrication: Pluronic F-127 as surfactant (5% in weight), GO (0.005%, 0.01% and 0.05% in weight) and TPP (0.005%, 0.1%, 0.5% and 1% in weight). The 0.1% loading of the photosensitizer was chosen to be combined with: (i) the addition of the surfactant, (ii) the addition of GO 0.01% and (iii) the addition of both the surfactant and GO 0.01%, in order to explore the effect of the different additives on the morphology and porosity of the membranes and on their photophysical and phototoxicity properties.

### 3.2. Morphological characterization of the prepared membranes

**Fig. 1:**
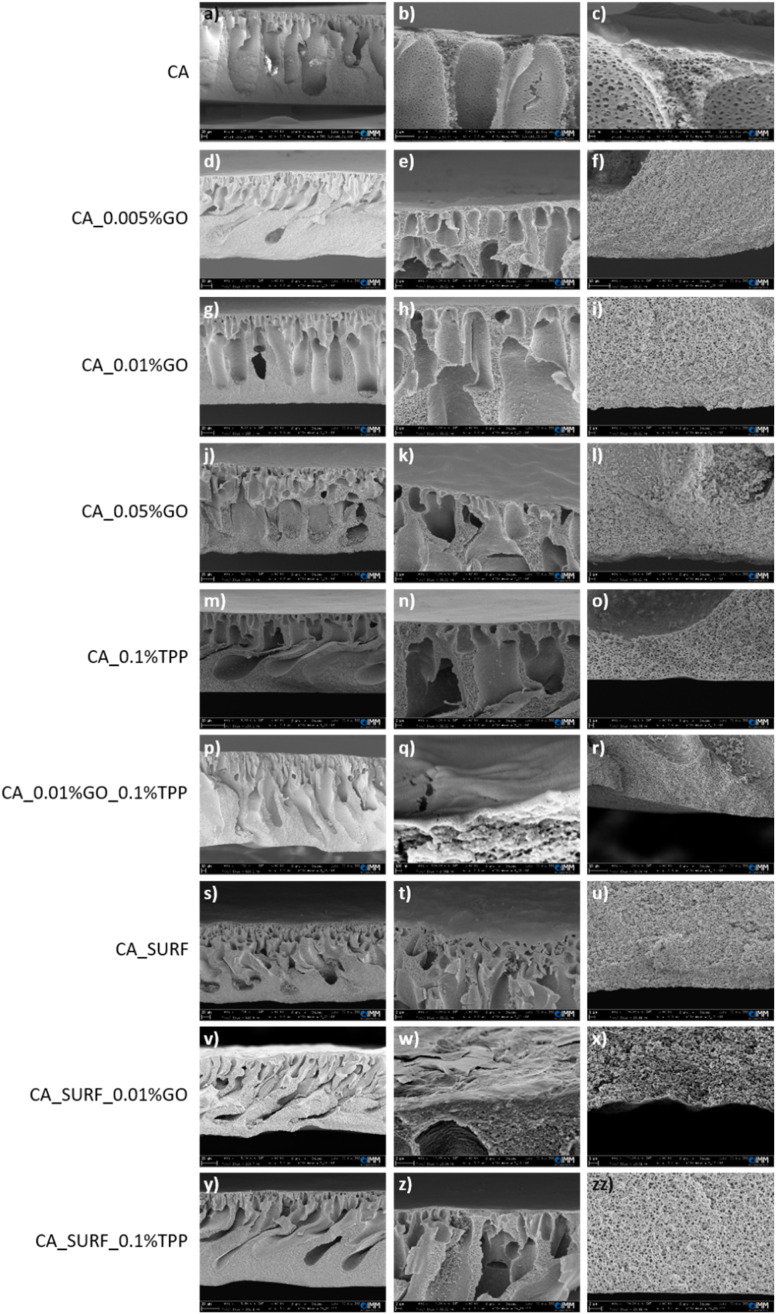
Different magnifications (higher from left to right) of cross-sections SEM images of bare cellulose acetate membranes and membranes modified with GO, TPP, surfactant and their mixtures. The scale bars are: (a, d, g, j, m, s, v and y): 20 μm; (f, p and r): 10 μm; (b, e, h, i, k, l, n, t, w, z and zz): 2 μm; (o, u and x): 1 μm; (c): 200 nm; (q): 100 nm.

In order to evaluate the effect of introduction of the different additives in the polymer solution on the final porous structure of the prepared membranes, the cross-section morphology of the most significant membranes has been investigated by means of SEM analysis. Fig. 1 shows the SEM cross-section images of the membranes. It can be observed that they possess macro-voids and finger like anisotropic structures and, upon high magnification, the presence of a fine porous network structure is evidenced. The incorporation of the three additives and their mixtures does not have a significant influence on the microscopic cross-sectional structure of the modified membranes.

**Fig. 2:**
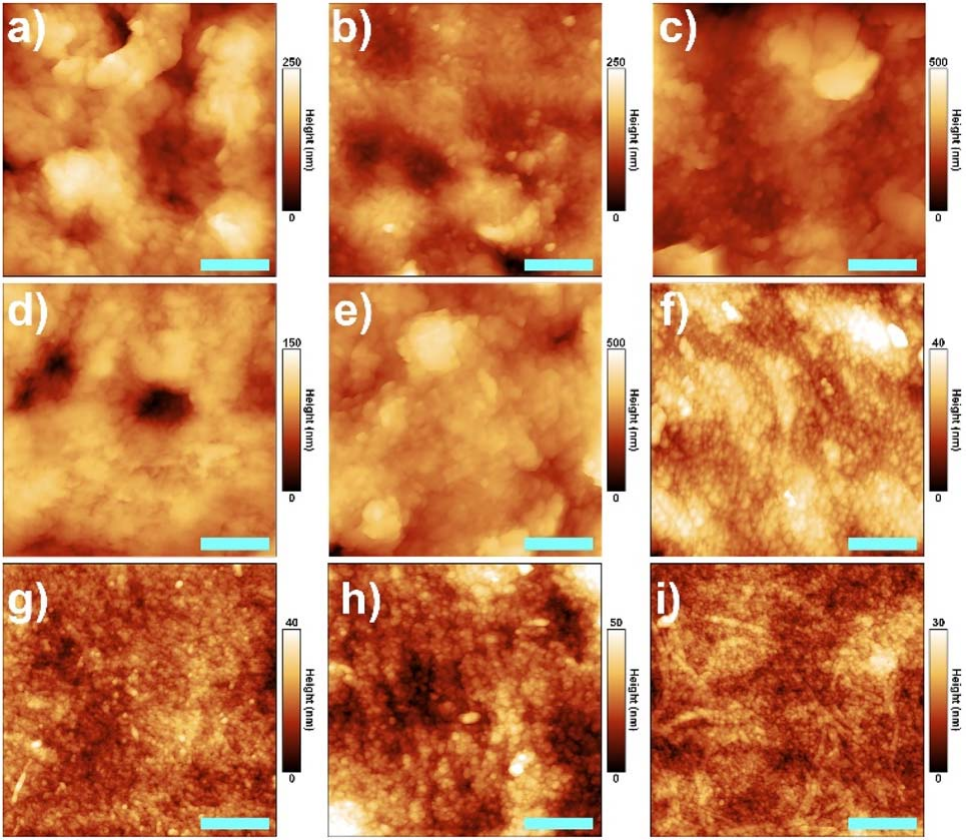
AFM images with a scale bar of 500 nm of (a) CA_0.005% GO, (b) CA_0.01% GO, (c) CA_0.05% GO, (d) CA_0.1% TPP, (e) CA_0.01% GO_0.1% TPP, (f) CA_SURF, (g) CA_SURF_0.01% GO, (h) CA_SURF_0.1% TPP, and (i) CA_SURF_0.01% GO_0.1% TPP.

To further characterize the effect of the different additives introduced in the membrane preparation on the characteristics of the membranes surface, AFM images were acquired for the prepared materials (Fig. 2). As summarized in Table 1, the roughness of membranes containing GO is significantly higher than that of bare CA (10 ± 2 nm) and increases as its percentage increases. Also, the addition of TPP leads to a small increase of roughness compared to the bare CA. However, this effect of both additives (GO and TPP) on membrane roughness is actually nullified when they are combined with the addition of the surfactant. The latter, in fact, produces an evident effect of smoothing of the membrane surface when present (Table 1).

**Table 1:** Summary of membrane surface roughness parameters as a function of the preparation conditions

Sample	Fig. 2	Surface roughness (nm)
CA		10 ± 2^a^
CA_0.005% GO	a	35 ± 5
CA_0.01% GO	b	40 ± 5
CA_0.05% GO	c	58 ± 5
CA_0.1% TPP	d	18 ± 5
CA_0.01% GO_0.1% TPP	e	50 ± 5
CA_SURF	f	6 ± 2
CA_SURF_0.01% GO	g	4 ± 2
CA_SURF_0.1% TPP	h	9 ± 2
CA_SURF_0.01% GO_0.1% TPP	i	4 ± 2

a From ref. 7.

### 3.3. Pore size and porosity

Pore size and porosity of the investigated membranes are reported in Table 2.

**Table 2:** Mean flow pore diameter and porosity of all the membranes

Sample	Mean flow pore diameter (nm)	Porosity (%)
CA	30 ± 5	74 ± 1
CA_0.005% GO	40 ± 10	77 ± 3
CA_0.01% GO	50 ± 10	76 ± 1
CA_0.05% GO	60 ± 10	77 ± 2
CA_0.1% TPP	30 ± 5	74 ± 2
CA_0.01% GO_0.1% TPP	50 ± 10	77 ± 1
CA_SURF	50 ± 10	77 ± 2
CA_SURF_0.01% GO	60 ± 10	78 ± 3
CA_SURF_0.01% GO_0.1% TPP	60 ± 10	77 ± 3

The membrane without the use of any additives shows the smallest pore size, equal to 30 nm, accompanied by the lowest value of porosity (74%). The average pore size for the other membranes ranges from 30 nm to 60 nm. In particular, the addition of GO, as well as of the surfactant, promoted the formation of membranes with a larger pore size (up to 60 nm). As already reported in literature, in fact, the addition of GO into dope solution increases the thermodynamic instability of the system which results in a pore size enlargement.^29,30^ This is also reflected in a slight porosity increase (from 74 to 77%).

The single addition of TPP did not alter the pore size and the porosity with respect to the bare CA membrane, while the addition of the surfactant in combination with GO resulted in membranes with a slightly higher pore size (60 nm) and porosity (78%) with respect to the membranes prepared with GO and without surfactant.

### 3.4. Optical characterization

#### 3.4.1. Photophysical properties of membranes loaded with TPP

**Fig. 3:**
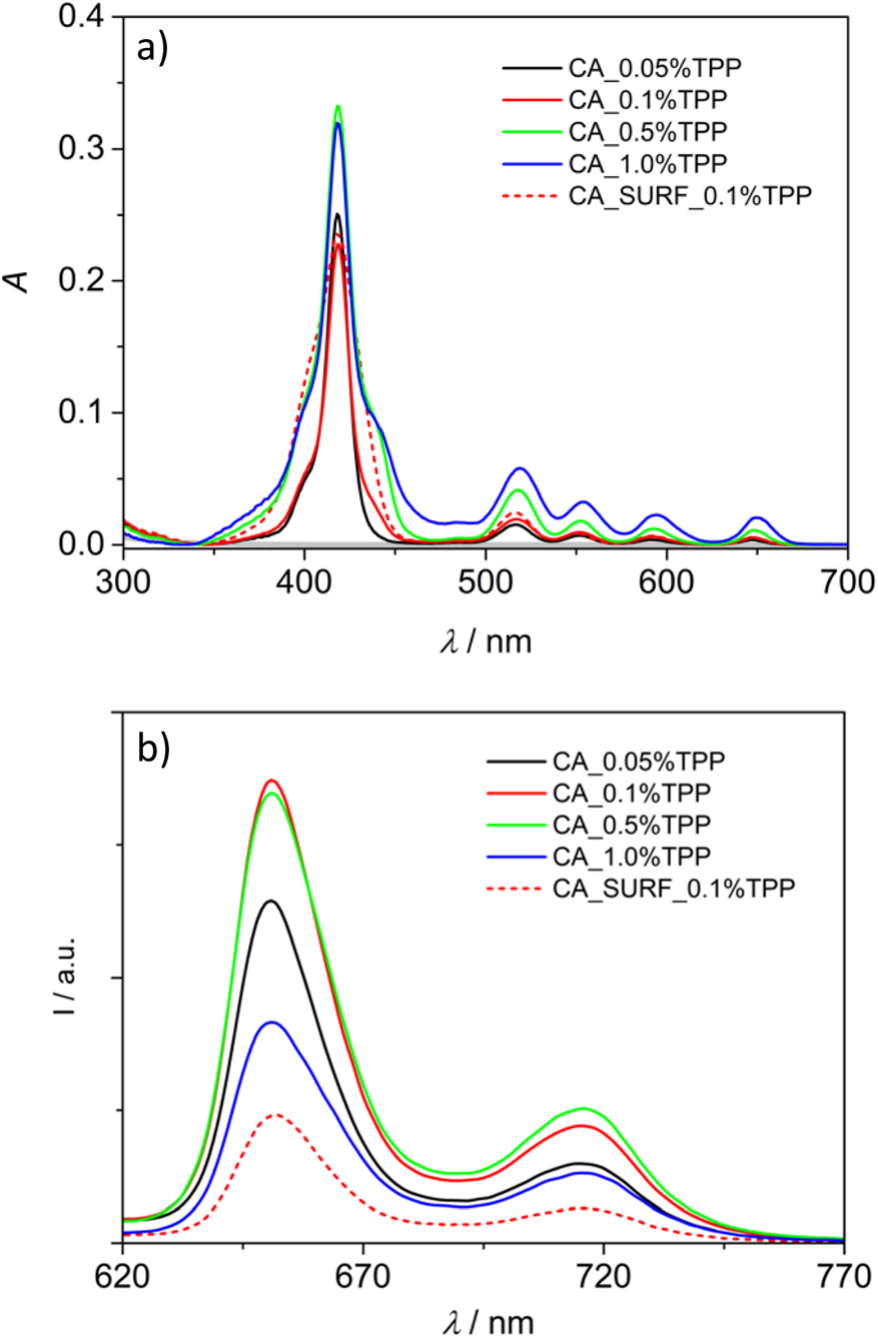
Absorption (a) and uncorrected emission (b) spectra of CA membranes with different loadings of TPP and with the combination of the surfactant and 0.1% TPP. The grey spectrum in (a) represents the absorption of the bare CA membrane. In (b) excitation at 510 nm.

Reflectance spectra of the membranes containing the photosensitizer in different loadings were collected by using a spectrophotometer equipped with an integrating sphere, and then converted in absorption spectra with the Kubelka–Munk function (see the Experimental section). Fig. 3a shows the derived spectra: it can be noticed that they well resemble that of TPP in solution^31,32^ (a comparison is shown in Fig. S1a†). A trend in the increase of the Q-bands (500–670 nm) intensity is observed upon increasing the TPP loading. It is accompanied by a broadening of the Soret band (350–450 nm) in the samples with >0.05% loading and in that combining 0.1% TPP and the surfactant. The broadening and splitting of the Soret band are evidenced by comparing the spectra normalized at 512 nm (Fig. S2†), and can be ascribed to exciton coupling between closely spaced porphyrin units in the membrane bearing the highest loading and in the presence of the surfactant that can create microenvironments where the dye aggregates inside the polymeric matrix.

Emission spectra have been collected, upon excitation at 510 nm, for all the membrane samples in the same experimental conditions, in order to derive a semi-quantitative comparison of the emission intensity; they are shown in Fig. 3b. It can be observed that the spectra display features typical of TPP emission^31,32^ (Fig. S1b†) and an intensity increase is observed from CA_0.05% TPP to CA_0.1% TPP. Conversely, the intensity is not further increasing for CA_0.5% TPP and is decreasing for CA_1.0% TPP, despite the higher absorption of these samples at 510 nm. An important quenching is also observed for the sample containing the surfactant. These data confirm the hypothesis that, for the highest loadings and in presence of the surfactant, the dye molecules aggregate inside the membranes, with the result of a static quenching of their emission. Normalization of the spectra at 716 nm reveals some changes in the relative intensity of the two vibronic bands along the series, with a decreased relative intensity of the band at 650 nm for the highest loadings (Fig. S3†), likely due to reabsorption effects. The emission spectrum of the surfactant containing membrane is red-shifted of 2 nm with respect to the other spectra, indicating a different environment experienced by the dyes in this sample, where the aggregation is probably more organized.

Excitation spectra, collected at 720 nm, indicate a good match with the porphyrin absorption in the Q-bands region for loadings up to 0.1%, while slight deviations and blue shift are detected for the samples CA_TPP 0.5% and CA_TPP 1.0% (Fig. S4†), ascribable to different absorption features of the emissive species in the aggregates.

Excited state lifetimes of the TPP molecules loaded into the membranes have been measured for all samples in bulk conditions with a front-face setup, and are reported in Table 3. All decays are characterized by a multi-exponential behaviour that can be reasonably treated with a biexponential fitting. Lifetimes of the order of 10–11 ns, typical of the monomeric TPP (and similar to that observed for drop-casted CA_TPP films, where a mono-exponential decay of 11.7 ns is detected) are accompanied by short lifetimes of the order of 0.3–1.0 ns (Table 3). The relative weight of the short components raises in the sample with the highest loading and in that with the surfactant, indicating an increase in aggregation phenomena.^33^ In the latter case, moreover, the long lifetime appears shorter than in the other samples (9.0 ns *vs.* 10–11 ns), confirming the different microenvironment created by the surfactant.

**Table 3:** Fluorescence lifetimes for TPP in the indicated membranes measured with the time correlated single photon counting (TCSPC) technique; excitation at 465 nm and emission collection at 650 nm and 720 nm (averaged lifetimes from the two emission wavelengths; in brackets: fractional intensities). FLIM fluorescence lifetimes with fractional intensity of the fitted sample area, excitation at 640 nm and emission centred at 715 nm

Sample	*τ*_TCSPC_ (ns) (*I*_*i*_%)	*τ*_FLIM_ (ns) (*I*_*i*_%)
CA_0.05% TPP	0.7 (28%); 10.2 (72%)	1.8 (13%); 8.1 (87%)
CA_0.1% TPP	0.4 (23%); 10.5 (77%)	1.9 (14%); 8.3 (86%)
CA_0.5% TPP	0.4 (16%); 11.0 (84%)	1.0 (2%); 10.4 (98%)
CA_1.0% TPP	0.3 (34%); 10.9 (66%)	0.5 (29%); 3.1 (11%); 10.0 (60%)
CA_SURF_0.1% TPP	1.0 (53%); 9.0 (47%)	0.7 (32%); 2.6 (13%); 9.0 (55%)

**Fig. 4:**
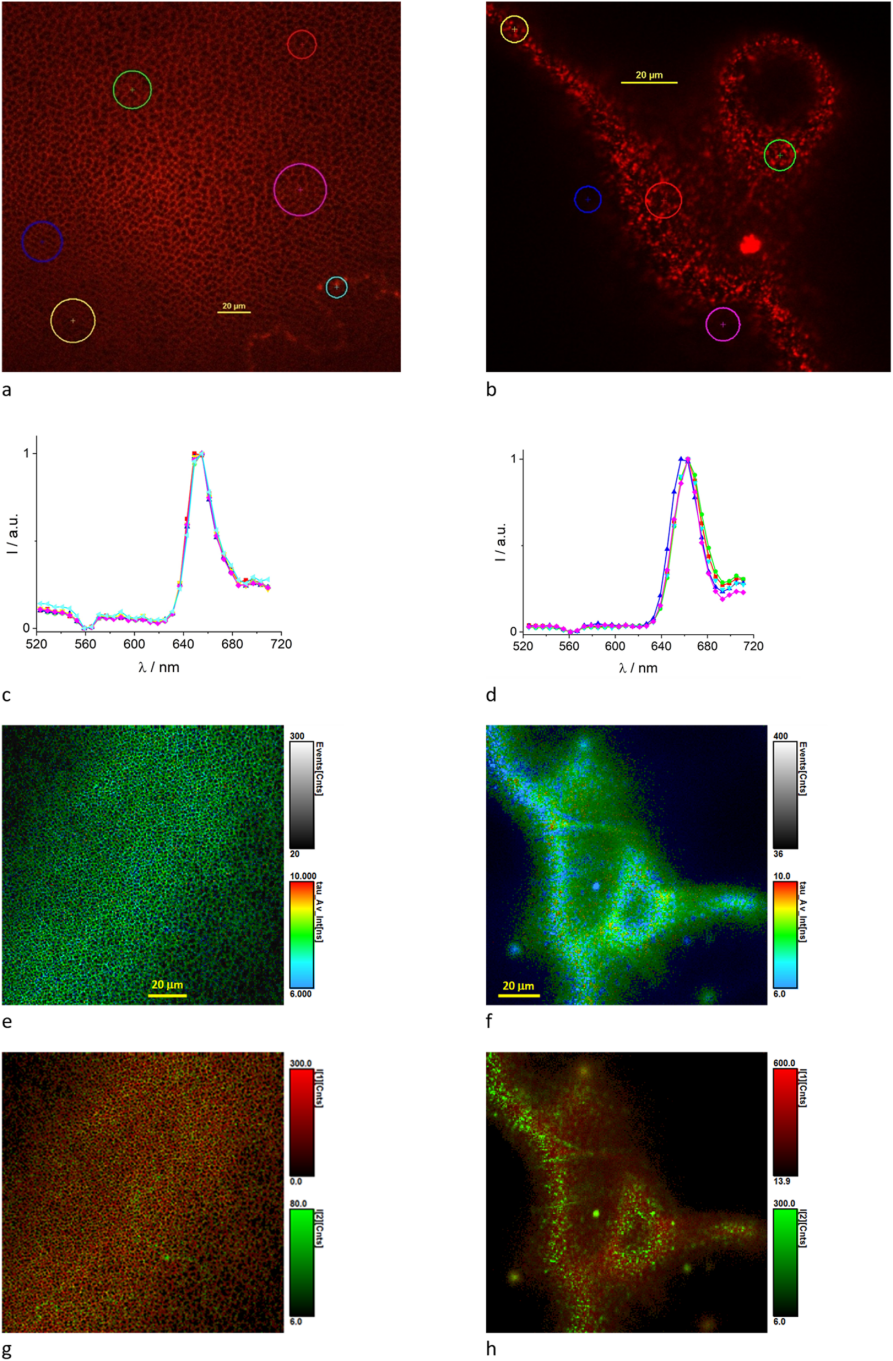
Confocal images of CA_0.1% TPP (a, c, e and g) and CA_1.0% TPP (b, d, f and h) obtained using 20× objective; (a and c) spectral image and spectra of ROI for excitation at 405 nm; (b and d) spectral image and spectra of ROI for excitation at 488 nm; (e–h) FLIM images for excitation at 640 nm and emission collected at 715 nm; (e and g) images showing the average fluorescence lifetime with color scale covering the 6–10 ns range; (f and h) images showing the intensity *I*_1_ and *I*_2_ for the long (red) and short (green) lifetime, respectively.

Further analysis of the membranes loaded with TPP was performed by means of confocal fluorescence imaging. In addition to fluorescence intensity imaging, also spectral and fluorescence lifetime imaging were exploited to characterize the membranes. Fig. 4 shows representative spectral images collected for the samples CA_0.1% TPP and CA_1% TPP. Excitation at 405 nm leads to observe almost exclusively the typical features of the TPP fluorescence spectrum with a maximum at 653 nm (Fig. 4c). Excitation at 488 nm gives a different spectrum, especially for the low TPP loading where the TPP fluorescence sums with blue-shifted fluorescence peaking at 550 nm circa, intrinsic of the CA membrane (Fig. S5a†). From the morphological point of view one can discern a membrane with dark non-emissive holes of different sizes, with diameters of 2–10 μm (Fig. 4a). In the case of the highest TPP loading (1%) we observe aggregates in correspondence of the holes of the membranes (Fig. 4b). Only in this case we observe a confocal fluorescence spectrum with maximum shifted to 662 nm for regions of interest (ROI) showing the aggregates^33^ (Fig. 4d). This feature did not emerge for the bulk measurements where spatial resolution is lost and large areas are examined. Time-resolved confocal fluorescence imaging (FLIM) was performed on the samples and the results are collected in Table 3. Samples were excited at 640 nm to avoid excitation of the membrane and emission collected with a bandpass filter at 715 nm, in correspondence of the second vibronic band of the TPP spectrum. We needed a bi-exponential decay fitting function affording a lifetime of *ca.* 9 ns, dominating the fluorescence, and one of *ca.* 2 ns. The long lifetime values are in line with the bulk measurements (Table 3), with a fractional intensity that is even higher in FLIM. This may be due to the FLIM setup where scattering can be avoided better compared to bulk measurements. FLIM, in fact, allows to collect photons from a thin selected section of the sample thus reducing contribution from scattered light. In the case of the sample CA_1.0% TPP the fit requires a tri-exponential decay function and the shortest lifetime becomes more important and its value decreases significantly. The FLIM images (Fig. 4f and h) clearly show the shorter average lifetime and the higher fractional intensity of the shortest lifetime in correspondence of the aggregates. This is in agreement with TPP aggregation, since a short lifetime has been reported for porphyrin aggregates.^33^ In the presence of the surfactant, we needed a tri-exponential function to fit the decay. Due to the higher fractional intensity of the short lifetime, the average lifetime of TPP is reduced significantly while the spectrum maximum does not change (Fig. S5b†). We do not observe aggregates but local TPP environment may be different in the presence of the surfactant.

**Fig. 5:**
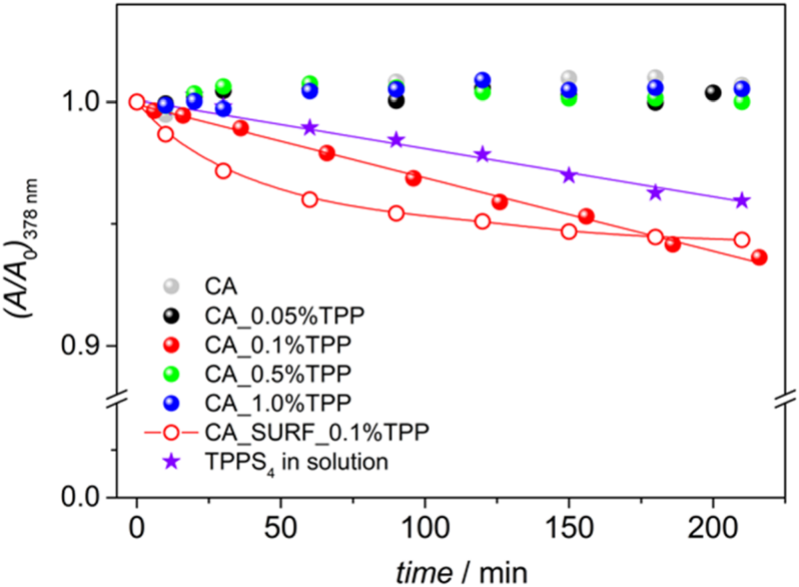
Variation of ADPA absorbance at 378 nm (fraction of the initial value) as a function of the irradiation time for the different membrane samples and for a mixed solution of ADPA and TPPS_4_. For membrane CA_0.1% TPP and TPPS_4_ in solution the lines represent linear fittings, while for CA_SURF_0.1% TPP the line is an indicative trend.

In order to assess the capacity of the membranes loaded with TPP to produce singlet oxygen, qualitative experiments were performed by making use of a singlet oxygen trap, anthracene-9,10-dipropionic acid (ADPA), which is consumed by the generated singlet oxygen. The measurements were conducted in heterogeneous conditions, by placing the membrane in a water solution of ADPA and irradiating the membrane, while immersed in the solution, at 512 nm for incremental time intervals (see the Experimental section for details). The consumption of ADPA in solution has been evaluated by the decrease of its absorption as a function of the irradiation time. The evolution of the spectra of ADPA solutions containing the membranes upon irradiation is reported in Fig. S6.† As a control, the bare CA membrane was tested and confirmed that, without the presence of the photosensitizers, the production of singlet oxygen is absent. A decrease in ADPA absorption is observable only for the membrane with 0.1% loading and for the membrane with 0.1% TPP and the surfactant. A plot reporting ADPA absorption at 378 nm as a function of the irradiation time is displayed in Fig. 5. The data suggest that the samples 0.1% TPP and SURF_0.1% TPP have a promising sensitization activity, displaying a different singlet oxygen release profile likely due to the different pore structure and dye distribution, as discussed above. The production of singlet oxygen could not be detected monitoring the ADPA absorbance for the membranes with the lowest and the highest TPP loadings; while for 0.05% TPP this can be ascribed to the low amount of photosensitizer in the membrane, for 0.5% TPP and 1.0% TPP a plausible explanation is the aggregation of the dyes that leads to triplet–triplet annihilation.

In order to validate the method, the standard 5,10,15,20-tetrakis(4-sulphonatophenyl)-porphyrin (TPPS_4_) has been used in solution, irradiating a mixed solution of ADPA and the standard in the same experimental conditions used for the membranes. TPPS_4_ is known to effectively produce singlet oxygen (*ϕ*_Δ_ = 0.64 in water)^34^ and, even if a direct comparison with the membrane behaviour is not possible due to the homogeneous conditions used for the standard, it can furnish indications on the effectiveness of the method and the conditions used. The spectra recorded for ADPA with TPPS_4_ in solution upon irradiation of the latter at 512 nm are shown in Fig. S7† and the decrease of ADPA absorbance at 378 nm is reported in Fig. 5. A linear correlation is observed and the profile of ADPA consumption is similar to that observed for the membrane CA_0.1% TPP, thus corroborating the method.

#### 3.4.2. Photophysical properties of membranes loaded with TPP and GO

Absorption and emission properties of membranes containing both GO and the photosensitizer have been analysed in order to assess if the presence of GO could affect the photophysical behaviour of the loaded dyes. 0.01% GO loading has been considered as the most promising modification in terms of membrane porosity, while 0.1% TPP was chosen as the best dye loading to impart significant absorption and emission features and singlet oxygen generation to the material. The further combination with the surfactant has also been explored.

The membranes were found to be rather inhomogeneous in terms of absorption and emission properties, and the spectra have been averaged over 4–5 measurements taken on different spots. Absorption spectra are shown in Fig. S8a,† together with the spectrum of the sample containing only 0.01% TPP as reference. The spectra appear quite similar on the Soret band, while important differences are observed on the Q-bands. Emission spectra, collected upon excitation at 510 nm (Fig. S8b†), reveal absence of quenching of TPP fluorescence when GO is present, while a significant quenching is detected when the surfactant is added, as observed in the CA membranes containing only the photosensitizer.

Excitation spectra collected at 720 nm (Fig. S9†) show features similar to those previously observed: a good match with the absorption bands in the Q region, except for slight deviations and blue-shift, and a poor match in the Soret band, where artefacts due to strong absorption are operative.

**Fig. 6:**
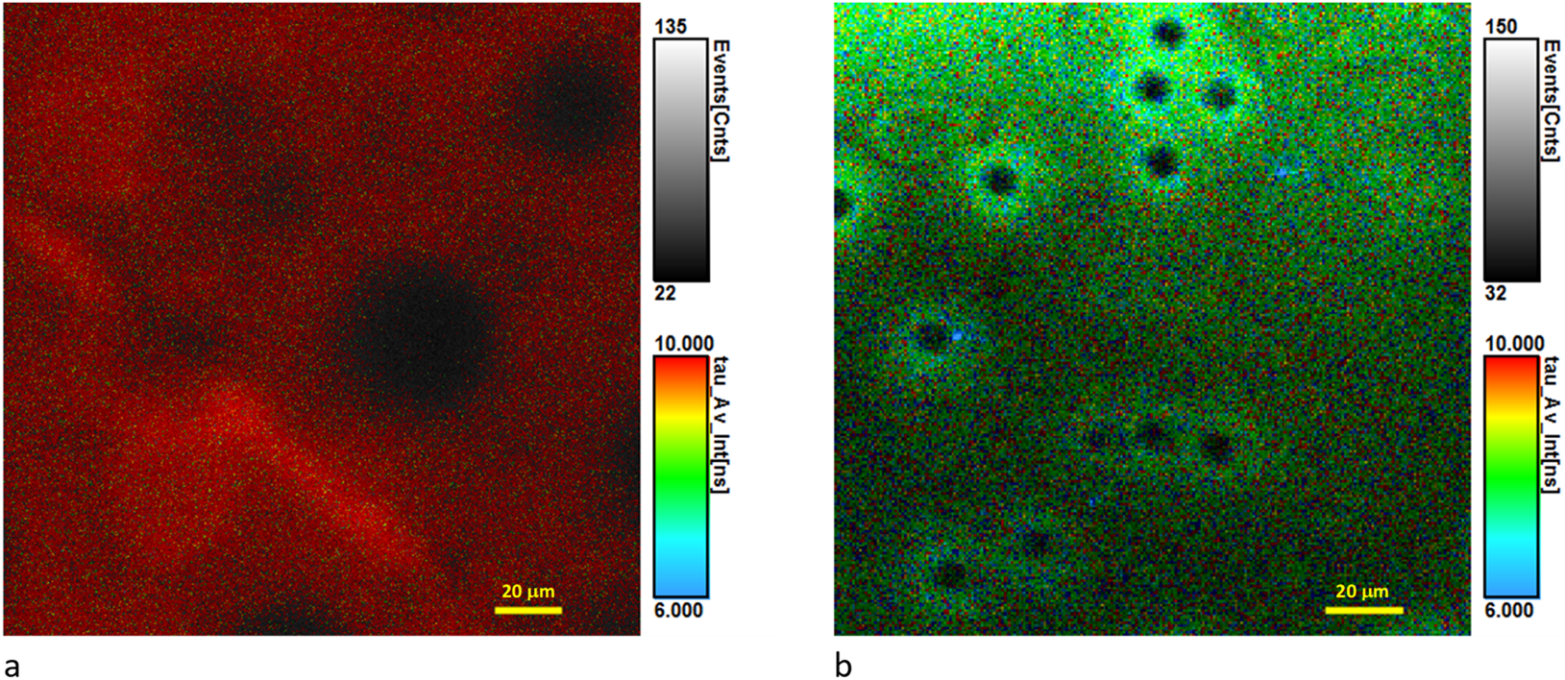
Confocal FLIM images of CA_0.01% GO_0.1% TPP (a) and CA_SURF_0.01% GO_0.1% TPP (b) obtained using 20× objective for excitation at 640 nm and emission collected at 715 nm; the images show the average fluorescence lifetime with color scale covering the 6–10 ns range.

Measured excited state lifetimes of the loaded dyes are presented in Table 4. For the membrane CA_0.01% GO_0.1% TPP the decay is almost mono-exponential with a lifetime of 11.5 ns, which is very similar to the lifetime measured in FLIM confirming the almost mono-exponential decay (Fig. 6). For the membrane containing the surfactant a bi-exponential decay is observed, with the long component of the order of 9 ns. In FLIM a tri-exponential fitting was required, with the long lifetime of 9 ns dominating the decay (Fig. 6). Confocal fluorescence spectra have maxima at 652 nm (Fig. S5c†). Again, the surfactant is likely changing the TPP local environment.

**Table 4:** Fluorescence lifetimes for TPP in the indicated membranes measured with the time correlated single photon counting (TCSPC) technique; excitation at 465 nm and emission collection at 650 nm and 720 nm (averaged lifetimes from the two emission wavelengths; in brackets: fractional intensities). FLIM fluorescence lifetimes with fractional intensity of the fitted sample area, excitation at 405 nm and emission centred at 655 nm

Sample	*τ*_TCSPC_ (ns) (*I*_*i*_%)	*τ*_FLIM_ (ns) (*I*_*i*_%)
CA_0.01% GO_0.1% TPP	≤0.1 (12%); 11.5 (88%)	0.5 (1%); 10.7 (99%)
CA_SURF_0.01% GO_0.1% TPP	0.4 (27%); 9.0 (73%)	0.4 (16%); 2.3 (15%); 9.1 (69%)

Overall the data show that the presence of GO is not affecting the photophysics of the TPP molecules inside the membrane, while the presence of the surfactant induces changes in the aggregation pattern, and also quenching, independently of the combination with GO.

It is interesting to note that samples containing 0.01% TPP in the presence of GO and surfactant are morphologically speaking different from the samples lacking the two additives (comparison of Fig. 4 and 6), with the distribution of the dye being more uniform and less reflecting the porosity of the membrane structure.

### 3.5. Antibacterial activity

The antibacterial activity of the fabricated membranes was assessed *in vitro* against two model strains, *S. aureus* and *E. coli*. Briefly, following the sedimentation of a well-defined concentration of bacteria (10^7^ cells) on the membrane coupon (*Ø* 6 mm) and, after the irradiation treatment, bacterial cells were mechanically detached from the membranes, and counting colony-forming units (CFU) was applied to estimate the antimicrobial effect. Analysis of colonies, in terms of number and size, was therefore performed after 24 hours of incubation at 37 °C.

**Fig. 7:**
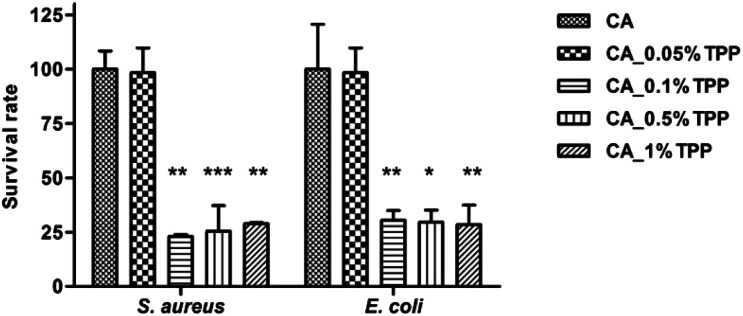
Photodynamic inactivation of *S. aureus* and *E. coli*. Bars indicate the percentage values (mean and standard deviation) of the colonies observed on the agar plates following the irradiation. CA_0.1% TPP, CA_0.5% TPP, and CA_1% TPP samples significantly decrease the viability of bacteria compared to dark controls.

The suitability of the experimental setting was first assessed by measuring the survival rate of bacteria when irradiated on the bare membrane (CA) (Fig. 7); no difference in CFU counting was observed between the unmodified sample material kept in the dark and following the irradiation step, thus excluding a reduction in bacterial viability due to the procedure. In addition, the experimental results indicated that CA membranes containing the TPP photosensitizer in different loadings (0.05%, 0.1%, 0.5%, 1%) with GO and/or SURF didn't impact with bacterial sedimentation on the materials, adhesion, and detachment from the materials as CFU counts of all dark controls did not differ with that obtained for the bare CA membrane (Fig. S10†). Thus, a chemically inert behaviour in absence of light was observed for all the tested materials.

As regards the photodynamic inactivation of *S. aureus* and *E. coli*, CA membranes loaded with 0.1%, 0.5%, and 1% TPP efficiently decreased the viability of strains reducing the number of colonies on the plate at the same extent, and without differences between Gram positive and Gram negative strains (Fig. 7). A similar level of susceptibility to photodynamic inactivation with porphyrin-conjugated cellulose materials of both groups of bacteria has been previously reported.^35^ It can be noticed that CA membranes loaded with 0.05% TPP allowed a full growth of bacteria with CFU counts close to those obtained for the unloaded CA, indicating that, in our experimental condition, 0.1% corresponded to the minimum percentage content of TPP inducing bacterial photodynamic inactivation. On the other hand, an increase of TPP loading up to 0.5% and 1% resulted in the same photoinactivation activity (Fig. 7), indicating that the presence of TPP aggregates observed for these membranes did not prevent the amount of singlet oxygen, produced by monomeric TPP molecules, to exert the bactericidal effect. The observed high sensitivity of bacteria to singlet oxygen is likely determined by their strong adhesion to the membrane surface, overcoming the drawback of the very short diffusion distance of singlet oxygen, of the order of 200 nm.^36,37^ The latter considerations also accounts for the apparent discrepancy among the absence of detection of singlet oxygen production for membranes CA_0.5% TPP and CA_1.0% TPP and their demonstrated photoinactivation activity.

**Fig. 8:**
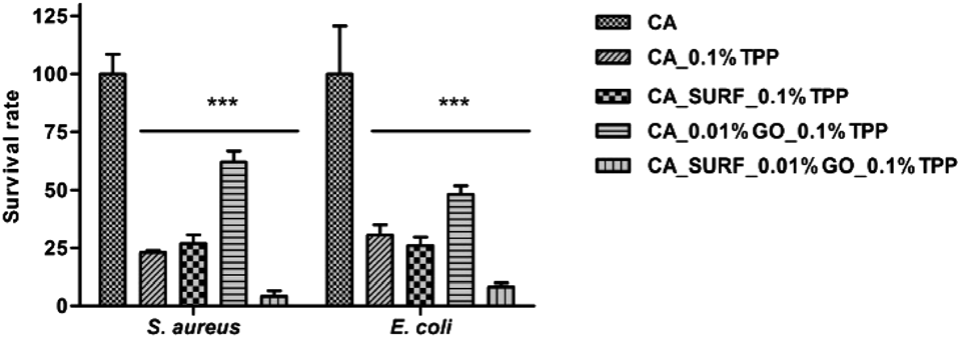
Photodynamic inactivation of *S. aureus* and *E. coli*. Bars indicate the percentage values (mean and standard deviation) of the colonies observed on the agar plates following the irradiation. The addition of the additives significantly decreases the viability of bacteria compared to the bare membrane (***p < 0.0001).

**Fig. 9:**
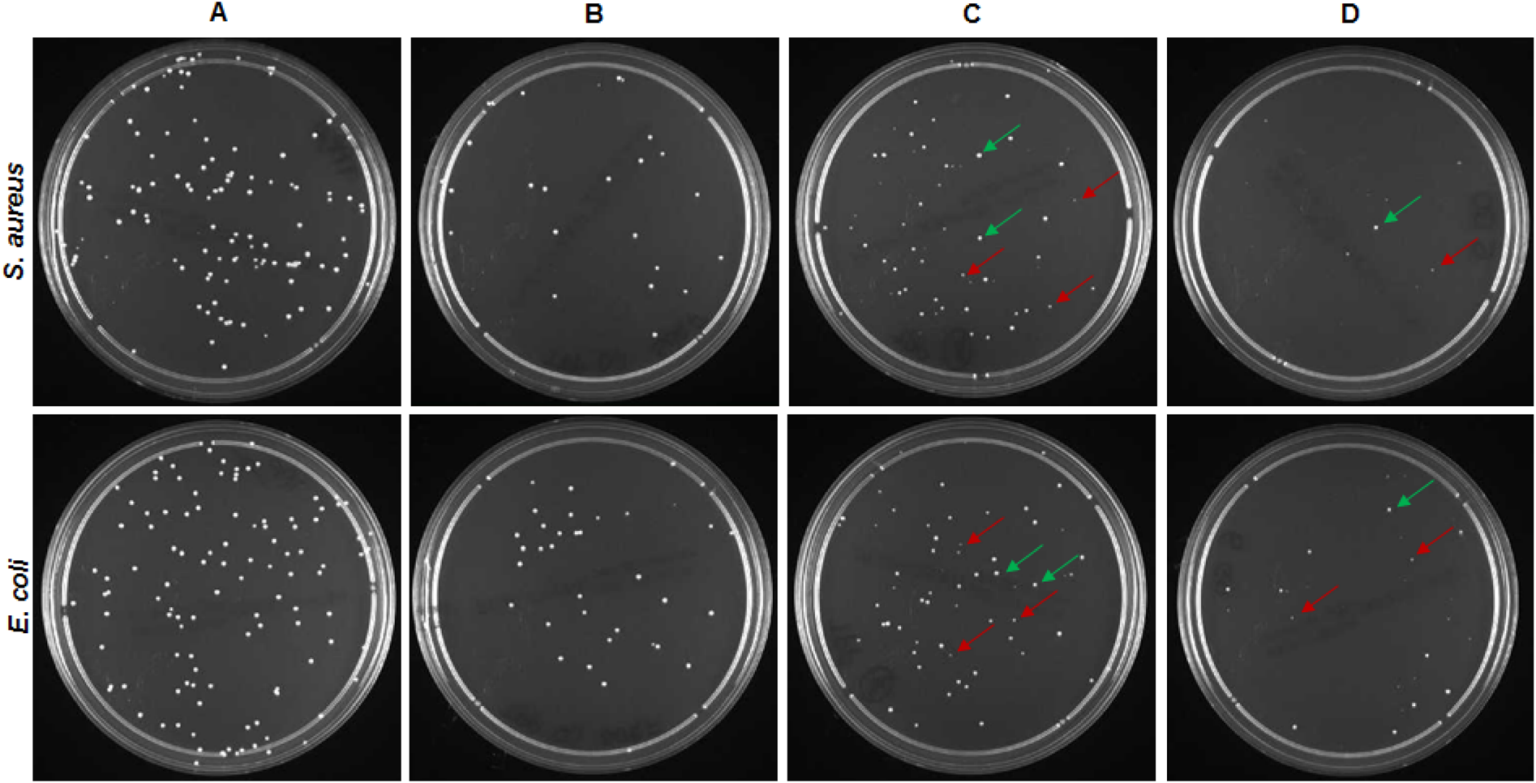
Colonies of *S. aureus* and *E. coli* treated with (A) CA as control, (B) CA_SURF_0.1% TPP, (C) CA_0.01% GO_0.1% TPP, and (D) CA_SURF_0.01% GO_0.1% TPP. Green arrows indicate CFUs with areas comparable to those grown in the control plates (1.35 ± 0.50 mm^2^); red arrows indicate CFUs smaller in size (0.15 ± 0.05 mm^2^).

The antibacterial studies were also performed on CA_0.1% TPP membranes containing SURF and/or 0.01% GO and results were evaluated in terms of number of CFUs (Fig. 8) and size of colonies produced on the agar plate after the photoinactivation treatment (Fig. 9). All samples significantly reduced bacterial viability as demonstrated by the survival rates of *S. aureus* and *E. coli* compared to the corresponding dark controls, thus demonstrating that the photosensitizer preserved its inhibitory potential in presence of the additives. No differences in the survival rates were observed comparing percentage values of CA_0.1% TPP and CA_SURF_0.1% TPP, for the two tested species.

As for CA membranes containing only GO, the relative high survival rates of 61.9% and 48.1% measured for *S. aureus* and *E. coli*, respectively, put out of sight the effect of this additive on bacterial growth evidenced in Fig. 9. Indeed, bacterial strains treated with CA_0.01% GO_0.1% TPP produced colonies with different sizes (Fig. 9C) that are all included in CFU counting. Of note, in this experimental condition, a significant proportion (up to 40%) of CFUs are smaller in size compared to colonies grown after treatment with CA_0.1% TPP and CA_SURF_0.1% TPP as well as in control samples. Finally, the highest antibacterial inactivation activity of >90% was obtained with CA membranes loaded with SURF and GO, suggesting a possible synergistic mechanism of activity of the membranes containing the three additives. Also in this case, small colonies appeared on the agar plates (Fig. 9D), confirming the effect of GO on bacterial proliferation.

Various mechanisms have been reported to explain the antibacterial activity of GO and GO-based materials, namely chemicals and physically induced in most of the studies. The bacterial inactivation caused by GO is related to oxidative stress, the cutting of intracellular metabolic routes and/or the rupture of the cell membrane resulting in a loss of cell membrane integrity, thus cell lysis. As expected, these effects are GO concentration- and time-dependent.^16,20^ It is therefore possible to speculate that, in our experimental conditions, the prevalent mechanism of activity of GO relies on the generation of reactive oxygen species, able to oxidize sensitive macromolecules, thus leading to inactivation of functional proteins, enzyme system disruption, amino acid metabolism disruption, and prevention of bacterial active transport. The resulting impaired bacterial metabolism, rather than a direct bactericidal effect, may generate the development of small colonies. This feature has been previously reported for other biomaterials having antibacterial activity *via* ROS production.^38^

It can be noticed that the membrane containing all the three additives, TPP, GO and the surfactant, showed the highest activity against both types of bacteria compared to the other experimental conditions (with significant differences associated to *p* < 0.0001). This result can be ascribed to the combination of photoinduced effects (production of singlet oxygen and other ROS) and morphological characteristics of the membrane imparted by the presence of the surfactant. In fact, the smoother surface and the more uniform distribution of TPP, evidenced by morphological and confocal analysis for the membranes containing the surfactant, could help in the bactericidal action of the produced ROS, which benefits from the closeness of the production site to the target pathogens.

## 4. Conclusions

Cellulose acetate membranes loaded with three different additives, *i.e.* a photosensitizer (TPP), graphene oxide (GO) and a surfactant (Pluronic F-127), have been prepared and fully characterised. The effect of the combination of the three additives has been analysed in terms of structural and morphological features and photophysical and photodynamic properties of the produced membranes. The photodynamic antibacterial activity has been assessed against two model strains, *S. aureus* and *E. coli*, demonstrating a photoinactivation rate higher than 90% for the membrane containing all the three additives. Moreover, the presence of GO induced a different growth of the bacteria, producing in some cases smaller colonies. These results have been discussed in terms of photoinduced activity (production of singlet oxygen and other reactive oxygen species) imparted by the presence of TPP and GO and distribution of the photosensitizer, related to the structural characteristics of the membrane, in turn determined by the presence of the surfactant. Overall, in this study, the careful control of the preparation conditions and the choice of the proper additives allowed the development of photoactive biocompatible polymeric membranes with excellent antibacterial activity, of vast potential in the field of bactericidal materials.

## Author contributions

Conceptualization: R. M., F. B., I. M., B. V.; methodology: R. M., B. V.; investigation: R. M., D. G., F. C., F. R., F. G., G. A. G, F. B., I. M., B. V.; data curation: D. G., F. R., F. G., G. A. G., F. B., I. M., B. V.; supervision and validation: V. M., A. F., F. B., B. V.; project administration, B. V.; writing – original draft: all authors; writing – review and editing: F. B., I. M., B. V.; funding acquisition: R. M., B. V.

## Conflicts of interest

There are no conflicts to declare.

## Supplementary Material

RA-013-D3RA04193J-s001
