# Supplementary material for: Cellulose acetate membranes loaded with combinations of tetraphenylporphyrin, graphene oxide and Pluronic F-127 as responsive materials with antibacterial photodynamic activity
Source: RSC Adv. 2023 Sep 7;13(38):26550–62. doi: 10.1039/d3ra04193j (PMC10483373; doi:10.1039/d3ra04193j)
Supplement: RA-013-D3RA04193J-s001 [file RA-013-D3RA04193J-s001.pdf]

Electronic Supplementary Material (ESI) for RSC Advances.
This journal is © The Royal Society of Chemistry 2023

Electronic Supplementary Information

Cellulose acetate membranes loaded with combinations of tetraphenylporphyrin, graphene oxide and Pluronic F-127 as responsive materials with antibacterial photodynamic activity

Rania E. Morsi,*[a,b] Denis Gentili,[c] Franco Corticelli,[d] Vittorio Morandi,[d] Alberto Figoli,[e] Francesca Russo,[e] Francesco Galiano,[e] Giovanna Angela Gentilomi,[f,g] Francesca Bonvicini,*[f] Ilse Manet*[b] and Barbara Ventura*[b]

[a] *Egyptian Petroleum Research Institute (EPRI), Nasr City, PO Box 11727, Cairo, Egypt.*
[b] *Institute for Organic Synthesis and Photoreactivity (ISOF), National Research Council (CNR), Via P. Gobetti 101, 40129 Bologna, Italy.*
[c] *Institute of Nanostructured Materials (ISMN), National Research Council (CNR), Via P. Gobetti 101, 40129 Bologna, Italy.*
[d] *Institute for Microelectronics and Microsystems (IMM), National Research Council (CNR), Via P. Gobetti 101, 40129 Bologna, Italy.*
[e] *Institute on Membrane Technology (ITM), National Research Council (CNR), Via P. Bucci 17/C, 87036 Rende (CS), Italy.*
[f] *Department of Pharmacy and Biotechnology, University of Bologna, Via Massarenti 9, 40138 Bologna, Italy.*
[g] *Microbiology Unit, IRCCS Azienda Ospedaliero-Universitaria di Bologna, Via Massarenti 9, 40138 Bologna, Italy.*

Table of Contents

Figure S1. Comparison between (a) molar absorption coefficient of TPP in toluene (orange, from Ref. 32, main text) and absorption of CA_0.05%TPP (black) normalized at 516 nm and (b) corrected emission spectra of TPP in toluene (orange, Ref. 32) and CA_0.05%TPP (black) normalized at 716 nm. — *Page S2*

Figure S2. Absorption spectra of CA membranes with different loadings of TPP and with the combination of the surfactant and 0.1% TPP, normalized at 512 nm. — *Page S3*

Figure S3. Uncorrected emission spectra of CA membranes with different loadings of TPP and with the combination of the surfactant and 0.1% TPP, normalized at 716 nm. Excitation at 510 nm. — *Page S3*

Figure S4. Excitation and arbitrarily scaled absorption spectra of CA membranes with different loadings of TPP and with the combination of the surfactant and 0.1% TPP. Emission collected at 720 nm. — *Page S4*

Figure S5. (a) Spectral image of CA_0.1%TPP excited at 488 with 6 nm resolution (OB 20×) and spectra of selected ROI; (b) Spectral image and FLIM image of CA_SURF_0.1%TPP and spectra of selected ROI for excitation at 405 and 488 nm; (c) Spectral images and spectra of selected ROI of CA_0.01%GO_0.1%TPP (left) and CA_SURF_0.01%GO_0.1%TPP (right) obtained using 20× objective for excitation at 488 nm. — *Pages S5-S6*

Figure S6. Absorption spectra of water solutions of ADPA containing the different membranes irradiated at 512 nm for the indicated times. — *Page S7*

Figure S7. (a) Absorption spectra of a water solution containing the standard $TPPS_4$ and ADPA upon irradiation at 512 nm and the spectrum of $TPPS_4$ at the same concentration as in the mixture (blue thick line). (b) The same spectra as in (a) subtracted by the contribution of $TPPS_4$; the spectral region above 400 nm is distorted by the subtraction of the intense Soret band, for this reason the variation of ADPA absorbance as a function of the irradiation time has been evaluated at 378 nm. — *Page S8*

Figure S8. Absorption (a) and uncorrected emission (b) spectra of CA membranes containing — *Page S8*

0.1% TPP and combinations of 0.1% TPP, 0.01% GO and the surfactant. In (a), the light grey and dark grey spectra represent the absorption of CA_0.01%GO and CA_SURF_0.01%GO membranes, respectively. In (b) excitation at 510 nm.

Figure S9. Excitation and arbitrarily scaled absorption spectra of the indicated membranes. Emission collected at 720 nm. *Page S8*

Figure S10. CFU counts of *S. aureus* and *E. coli* when the differently modified CA membranes were kept in the dark. Bars indicate the percentage values (mean and standard deviation) of the colonies grown on the agar plates compared to CA membrane control. *Page S9*

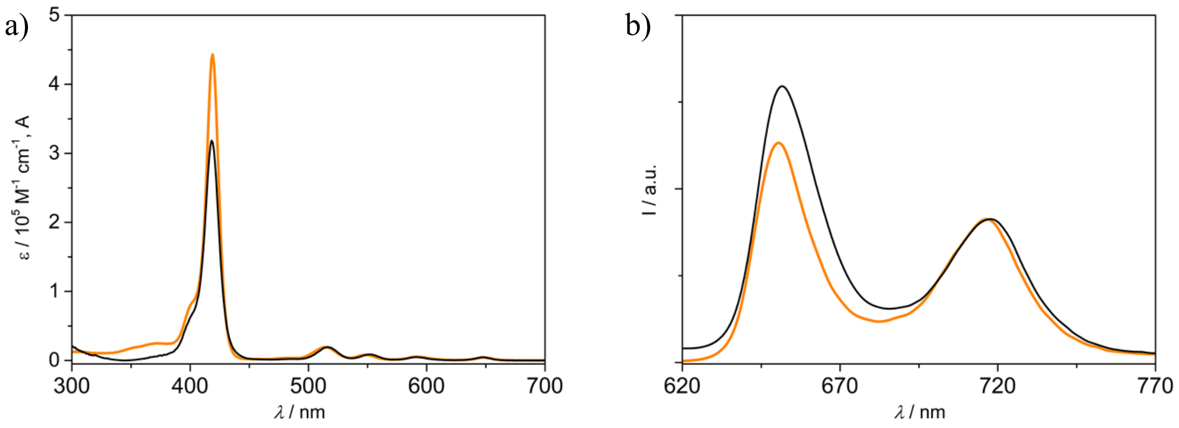

Figure S1. Comparison between (a) molar absorption coefficient of TPP in toluene (orange, from Ref. 32, main text) and absorption of CA_0.05%TPP (black) normalized at 516 nm and (b) corrected emission spectra of TPP in toluene (orange, Ref. 32) and CA_0.05%TPP (black) normalized at 716 nm.

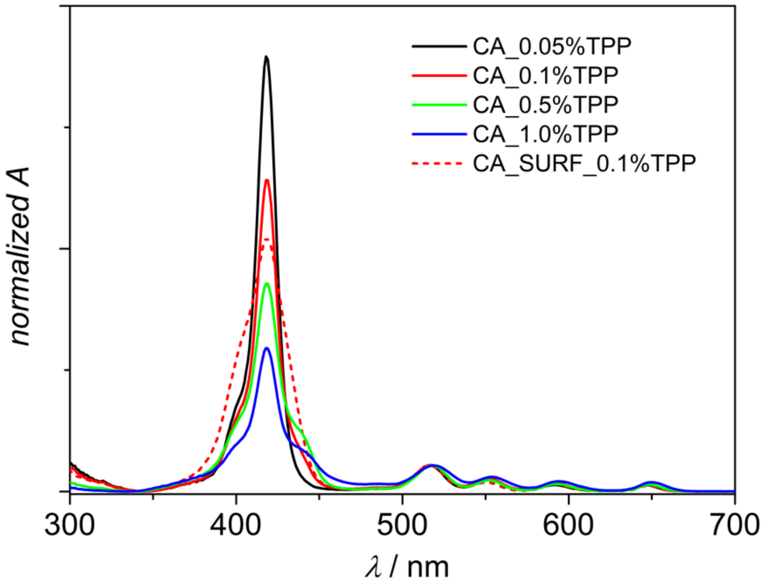

Figure S2. Absorption spectra of CA membranes with different loadings of TPP and with the combination of the surfactant and 0.1% TPP, normalized at 512 nm.

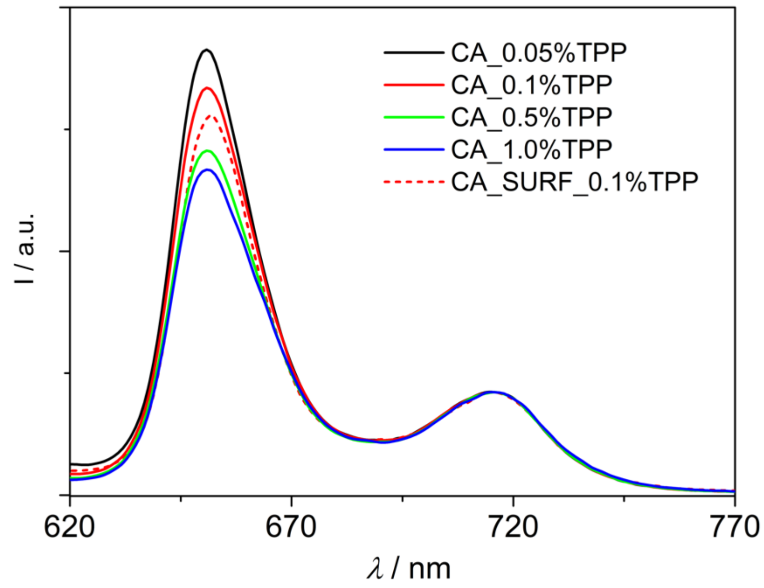

Figure S3. Uncorrected emission spectra of CA membranes with different loadings of TPP and with the combination of the surfactant and 0.1% TPP, normalized at 716 nm. Excitation at 510 nm.

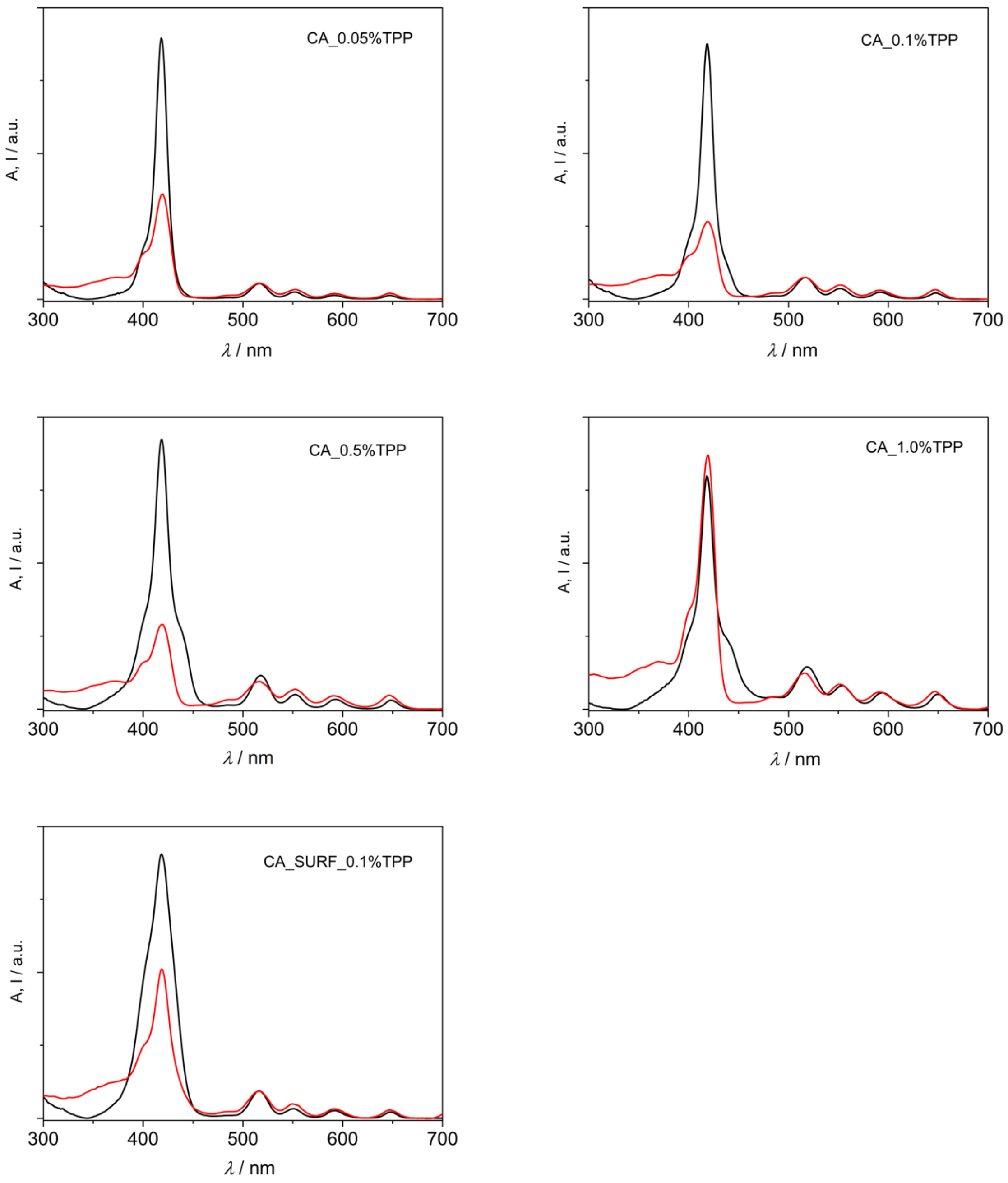

Figure S4. Excitation (red) and arbitrarily scaled (black) absorption spectra of CA membranes with different loadings of TPP and with the combination of the surfactant and 0.1% TPP. Emission collected at 720 nm.

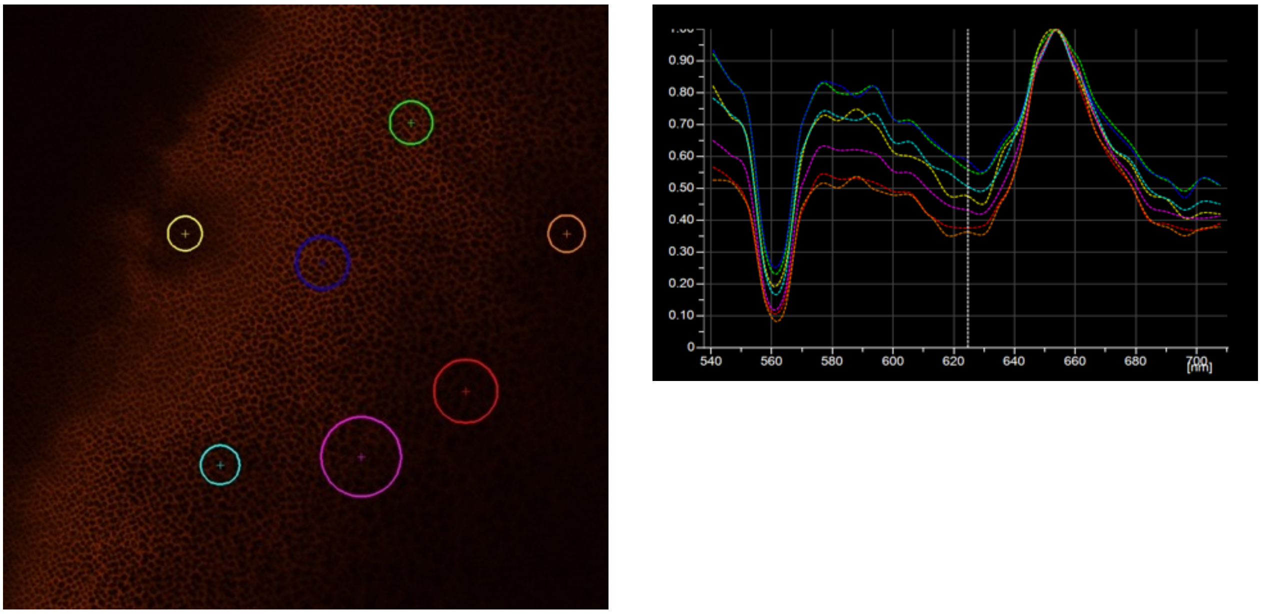
0.90
0.80
0.70
0.60
0.50
0.40
0.30
0.20
0.10
0
540
560
580
600
620
640
660
680
700
[nm]

a

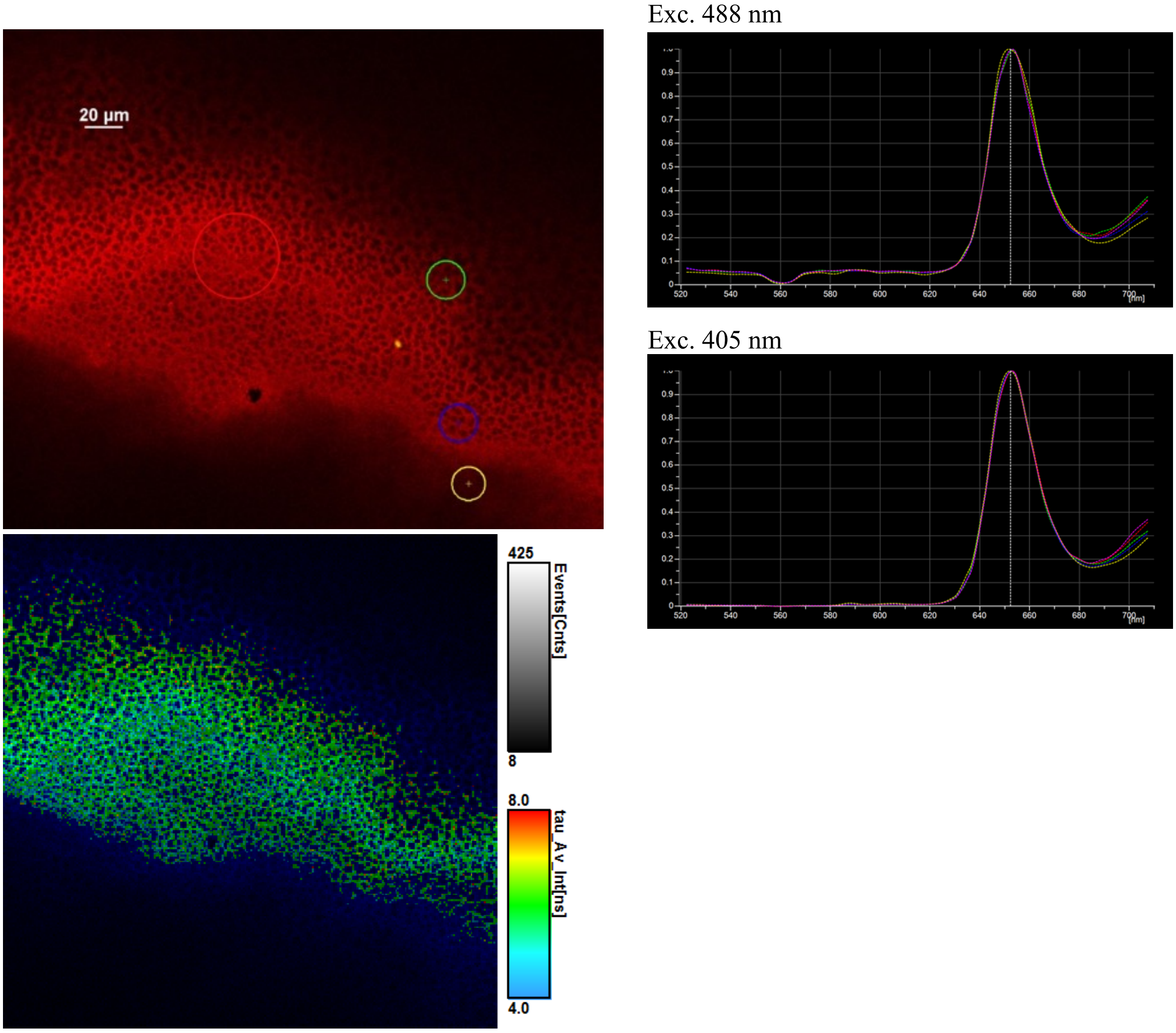
Exc. 488 nm
Exc. 405 nm
20 µm
425
Events[Cnts]
8
8.0
tau_Av Int[ns]
4.0

b

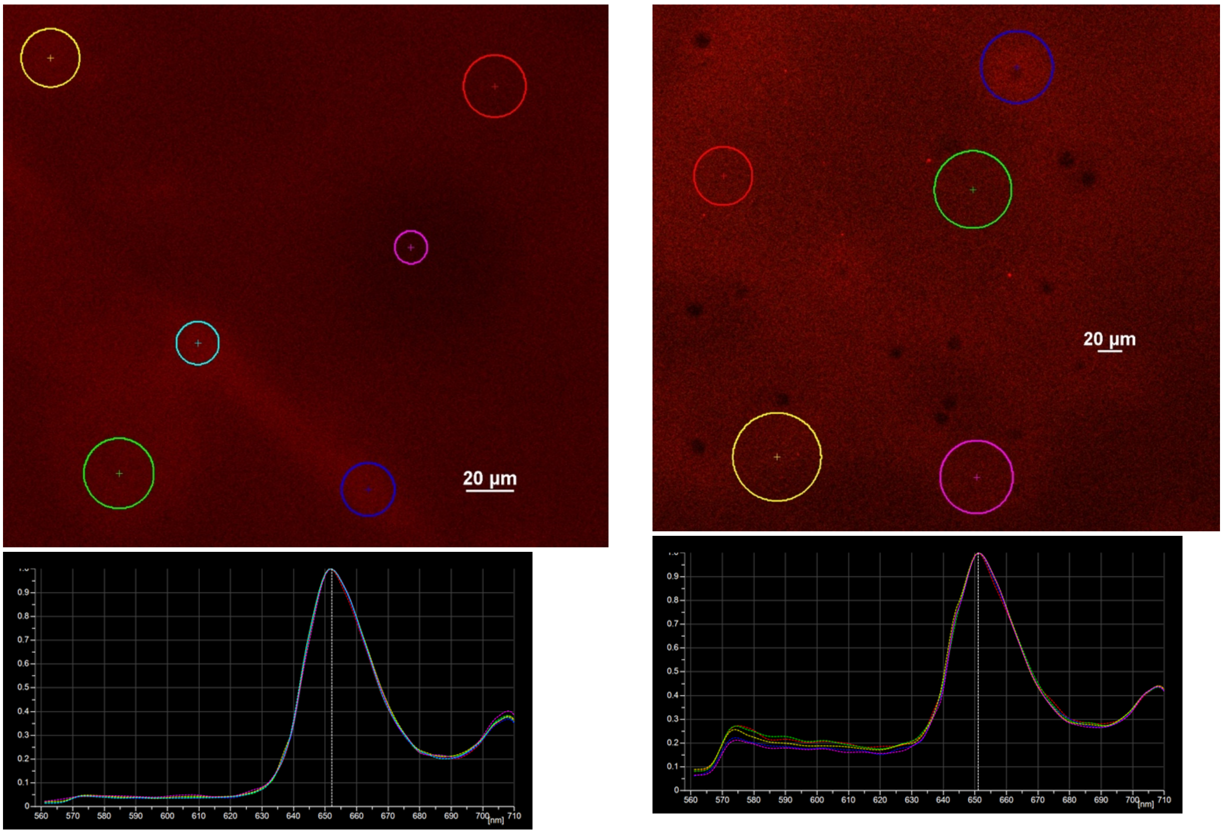

c

Figure S5. (a) Spectral image of CA_0.1%TPP excited at 488 with 6 nm resolution (OB 20×) and spectra of selected ROI; (b) Spectral image and FLIM image of CA_SURF_0.1%TPP and spectra of selected ROI for excitation at 405 and 488 nm; (c) Spectral images and spectra of selected ROI of CA_0.01%GO_0.1%TPP (left) and CA_SURF_0.01%GO_0.1%TPP (right) obtained using 20× objective for excitation at 488 nm.

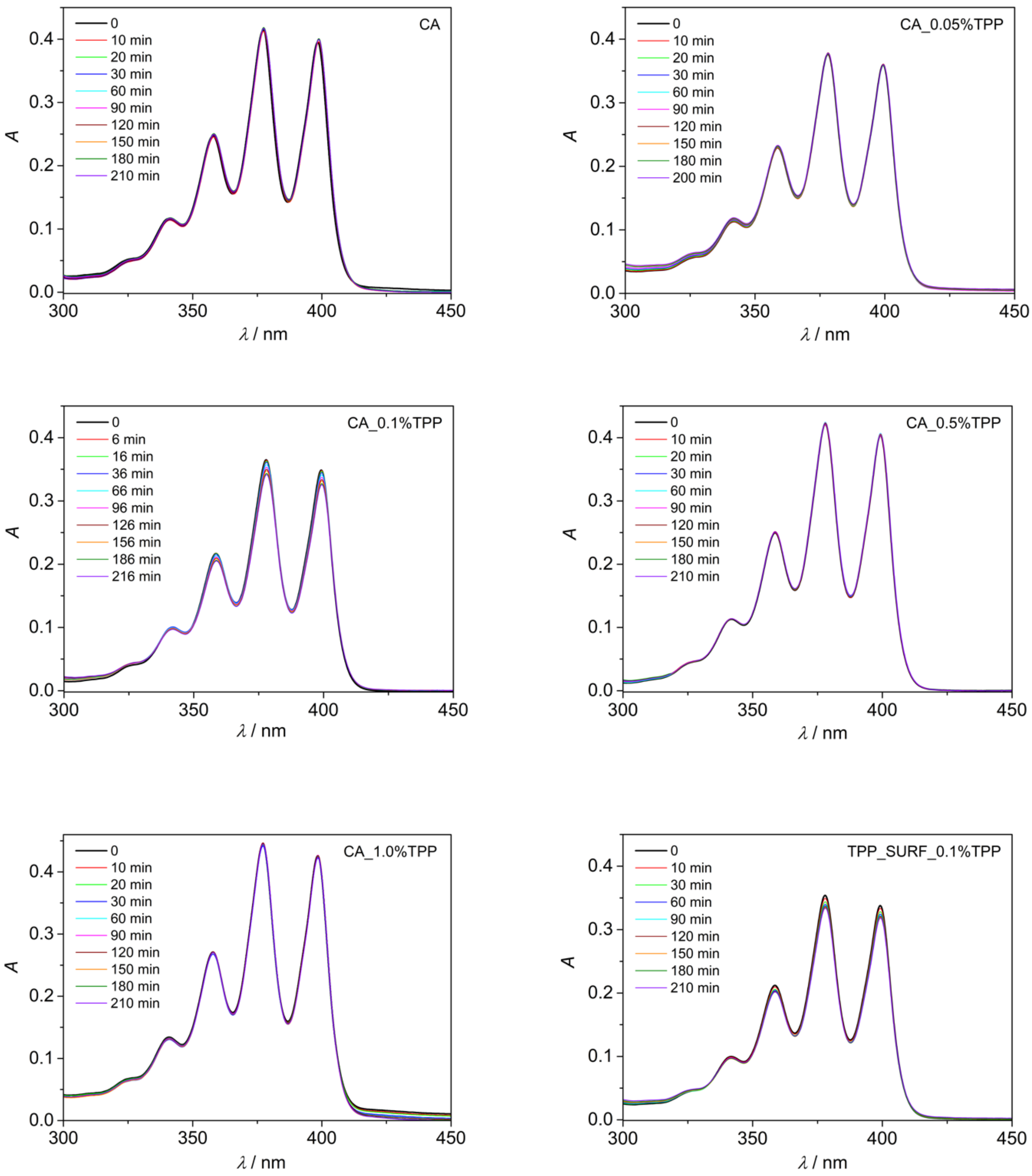

Figure S6. Absorption spectra of water solutions of ADPA containing the different membranes irradiated at 512 nm for the indicated times.

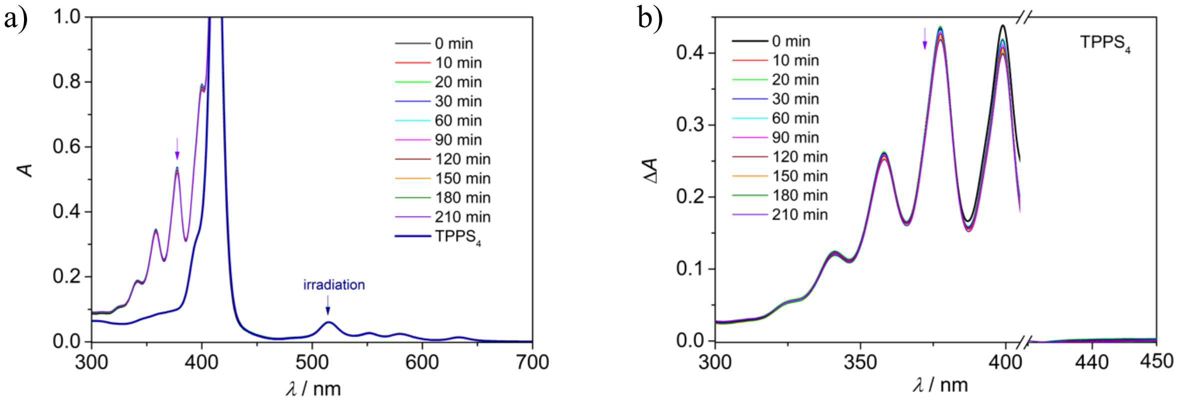

Figure S7. (a) Absorption spectra of a water solution containing the standard $TPPS_4$ and ADPA upon irradiation at 512 nm and the spectrum of $TPPS_4$ at the same concentration as in the mixture (blue thick line). (b) The same spectra as in (a) subtracted by the contribution of $TPPS_4$; the spectral region above 400 nm is distorted by the subtraction of the intense Soret band, for this reason the variation of ADPA absorbance as a function of the irradiation time has been evaluated at 378 nm (see Figure 5, main text).

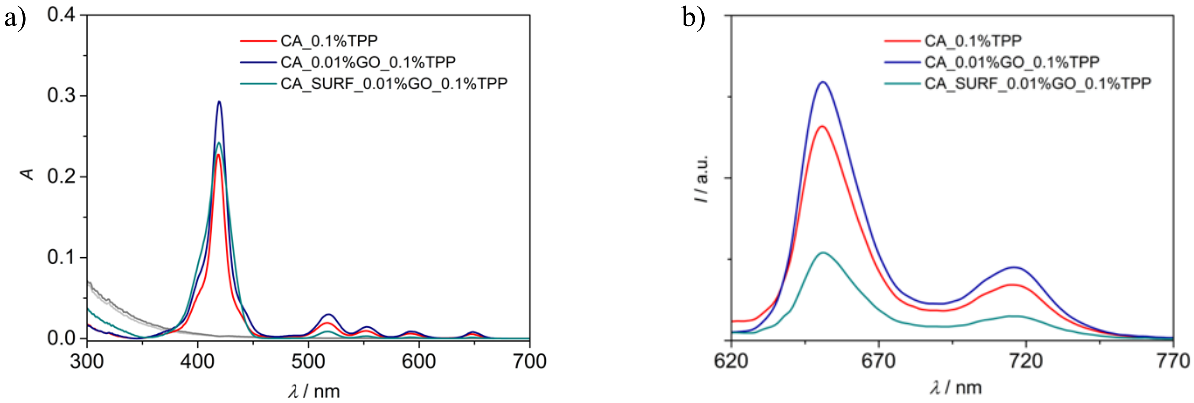

Figure S8. Absorption (a) and uncorrected emission (b) spectra of CA membranes containing 0.1% TPP and combinations of 0.1% TPP, 0.01% GO and the surfactant. In (a), the light grey and dark grey spectra represent the absorption of CA_0.01%GO and CA_SURF_0.01%GO membranes, respectively. In (b) excitation at 510 nm.

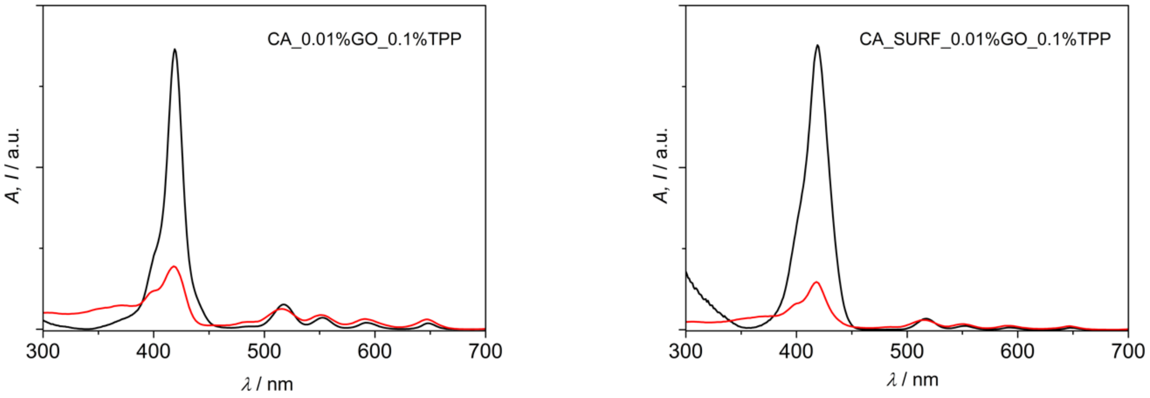

Figure S9. Excitation (red) and arbitrarily scaled (black) absorption spectra of the indicated membranes. Emission collected at 720 nm.

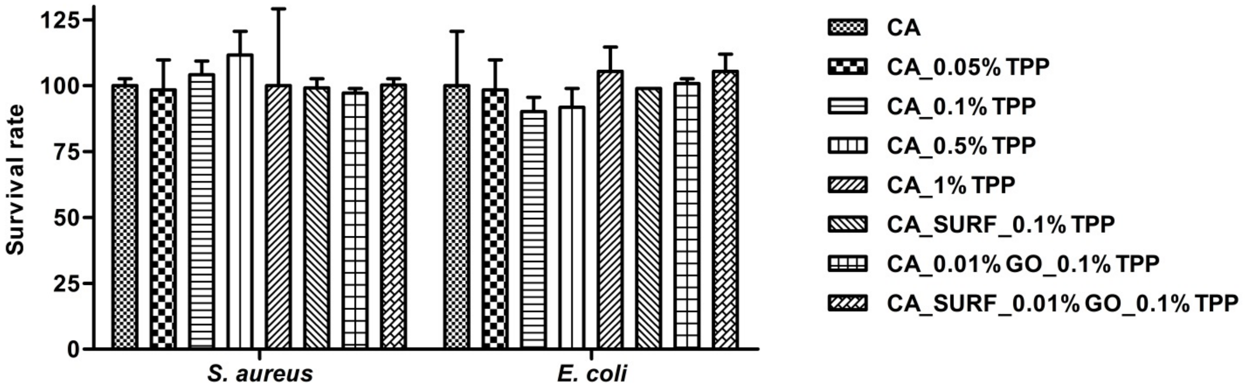

Figure S10. CFU counts of *S. aureus* and *E. coli* when the differently modified CA membranes were kept in the dark. Bars indicate the percentage values (mean and standard deviation) of the colonies grown on the agar plates compared to CA membrane control.
